# Identifying target processes for microbial electrosynthesis by elementary mode analysis

**DOI:** 10.1186/s12859-014-0410-2

**Published:** 2014-12-30

**Authors:** Frauke Kracke, Jens O Krömer

**Affiliations:** Centre for Microbial Electrosynthesis, The University of Queensland, Level 4, Gehrmann Laboratories Building (60), Brisbane, QLD 4072 Australia; Advanced Water Management Centre, The University of Queensland, Brisbane, QLD Australia

**Keywords:** Cathode, Electro synthesis, Bio production, Mediator, NAD/NADH, Extracellular electron transport, Electro fermentation, Anaerobic fermentation

## Abstract

**Background:**

Microbial electrosynthesis and electro fermentation are techniques that aim to optimize microbial production of chemicals and fuels by regulating the cellular redox balance via interaction with electrodes. While the concept is known for decades major knowledge gaps remain, which make it hard to evaluate its biotechnological potential. Here we present an *in silico* approach to identify beneficial production processes for electro fermentation by elementary mode analysis. Since the fundamentals of electron transport between electrodes and microbes have not been fully uncovered yet, we propose different options and discuss their impact on biomass and product yields.

**Results:**

For the first time 20 different valuable products were screened for their potential to show increased yields during anaerobic electrically enhanced fermentation. Surprisingly we found that an increase in product formation by electrical enhancement is not necessarily dependent on the degree of reduction of the product but rather the metabolic pathway it is derived from. We present a variety of beneficial processes with product yield increases of maximal 36% in reductive and 84% in oxidative fermentations and final theoretical product yields up to 100%. This includes compounds that are already produced at industrial scale such as succinic acid, lysine and diaminopentane as well as potential novel bio-commodities such as isoprene, *para*-hydroxybenzoic acid and *para*-aminobenzoic acid. Furthermore, it is shown that the way of electron transport has major impact on achievable biomass and product yields. The coupling of electron transport to energy conservation could be identified as crucial for most processes.

**Conclusions:**

This study introduces a powerful tool to determine beneficial substrate and product combinations for electro-fermentation. It also highlights that the maximal yield achievable by bio electrochemical techniques depends strongly on the actual electron transport mechanisms. Therefore it is of great importance to reveal the involved fundamental processes to be able to optimize and advance electro fermentations beyond the level of lab-scale studies.

**Electronic supplementary material:**

The online version of this article (doi:10.1186/s12859-014-0410-2) contains supplementary material, which is available to authorized users.

## Background

Metabolic redox limitations can be a crucial factor determining the viability of an industrial biotechnology process [1]. It could be shown, that increasing the amount of redox cofactors such as NADH or NADPH available to the microorganisms is an effective way to increase the product yield of reduced products such as propane [2,3] and also of commonly produced feed amino acids, e.g. lysine [4]. One novel and very promising approach to optimize the cellular redox state for production is to stimulate the metabolism electrically and therefore direct electron flow to desired products. The technique, termed microbial electrosynthesis or electro fermentation, shows potential to increase the efficiency of microbial production by providing additional electron donors or acceptors to the cells [5,6]. Even though nowadays already discussed as revolutionising future technology, little is known about its true potential as the fundamental processes still remain unclear [7,8].

Before general process design steps can be approached, a better understanding of the overall net benefits of possible target processes is needed [7,9]. These need to feature the production of a higher value carbon-body from a ubiquitous available cheap source by the investment of a reasonable amount of electric energy. While the first published electrically enhanced fermentations display in general proof-of-concept-studies, interesting substrate and product combinations still have to be investigated. The biggest challenge to drive microbial electrochemical technologies beyond fundamental studies is the optimisation of the microbial catalyst. Therefore the actual metabolic processes of microbe and electrode interaction need to be unveiled as they will not only decide about the choice of organism but might also play an important role for the achievable process benefit.

The research field of microbial fuel cells studies microbe-electrode interactions for many years. While key reactions could be identified a thorough understanding of the metabolic response to electrical enhancement has not been achieved yet [10,11]. The focus of microbial electrosynthesis especially requires more knowledge about cathodic electron transport and extracellular electron transfer (EET) capabilities of model organisms for production such as *E. coli*.

For anodic EET of electrogens such as *Geobacter* and *Shewanella* two main mechanisms are identified: direct electron transfer, which is performed by direct contact between the electrode surface and cellular components of the outer membrane (usually cytochromes), and indirect EET, which includes all forms of electron transfer between electrode and organisms mediated by soluble electron carrier molecules [12,13]. First studies on the cathode confirmed the possibility of donating additional electrons to the microbial metabolism by both EET mechanisms and its potential to increase production [5,14-17]. But it was also found that the involved mechanisms for electron uptake differ significantly from the known electron donating mechanisms [10,18,19].

Regardless of the major carbon metabolic pathway the effect of electrical enhancement is typically assumed to result in an increase or decrease of intracellular redox factors such as NADH or NADPH [8,11]. Electron transfer towards an anode is assumed to be coupled to energy conservation where the electrode functions as solid final acceptor during respiration [20,21]. However the exact ratio of electrons and protons that are transported remains purely speculative. Furthermore, it is not known by which mechanisms non-metal-respiring organisms might transfer electrons to an anode and whether that transfer promotes ATP generation or not [22]. Even though there is even less information available about cathodic electron transfer there is a general concept proposed that assumes the creation of a proton motive force by intracellular electron consumption, which is available for ATP synthesis [5,11,23,24]. In mediated electrically enhanced fermentations of *Actinobacillus succinogenes* Park and Zeikus observed an electron flow from the cathode into the product succinate [25]. Simultaneously, the electron transfer via the reduced mediator Neutral Red and the proton-pumping fumarate reductase complex of *A. succinogenes* induced proton translocation and therefore increased ATP synthesis [26]. While the activity of the proton pumping fumarate reductase of *A. succinogenes* is most likely solely responsible for the reported increase in proton flux through the ATPase complex, nowadays the theory about cathodic EET generally assumes that all electrons supplied by EET enter the cytoplasm as negative charge and catalyse intracellular, proton consuming reductions. Simultaneously, the proton consumption would lead to a proton gradient across the inner membrane that drives ATP synthesis [5]. But is this the only possibility? Observed is poor growth in very thin biofilms on cathodes [15,18], which seems to be surprising if cathodic EET could deliver redox power as well as energy (NADH and ATP). So the questions are: Are the protons involved in cathodic electron transfer generally available for ATP generation? And does electron transfer towards an anode always occur by the respiratory chain which thereby creates a proton gradient? What other ways of EET could occur and how would this impact production?

**Figure 1:**
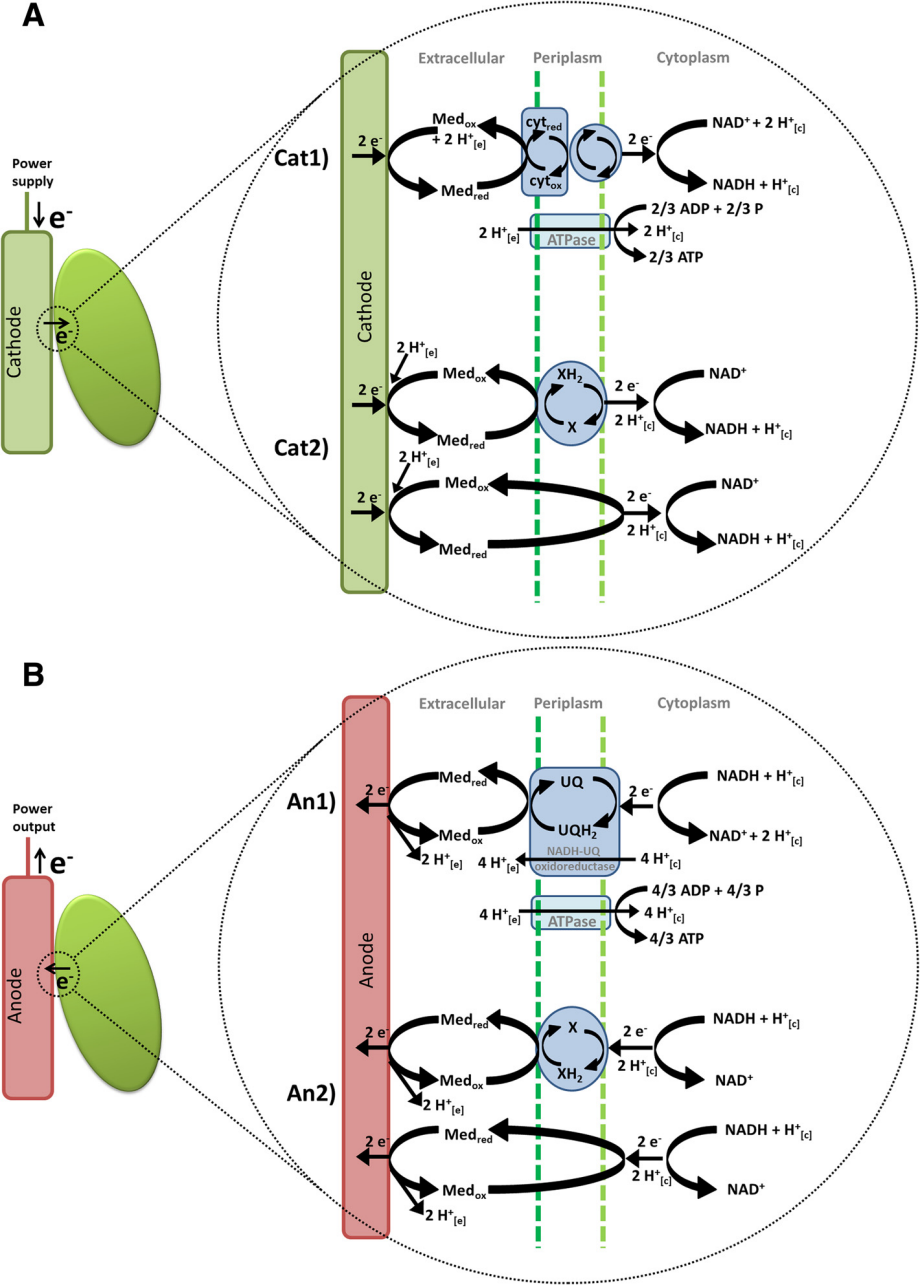
**Models for extracellular electron transport mechanisms coupled and uncoupled to energy conservation. (A)** Schematic image of two different electron transport mechanisms between cathodes and the microbial metabolism analysed within this study. *Cat1*) Electron transport via a mediator and a cascade of membrane bound complexes (e.g. cytochromes) with simultaneous ATP generation; *Cat2*) Direct reduction of NAD to NADH by electrons and protons by membrane bound enzymes (e.g. hydrogenases) or diffusion of the mediator molecule. **(B)** Two different models for microbial interaction with an anode as electron sink. *An1*) Electrons from the quinone pool are transferred to the electron mediator by membrane-bound enzymes such as NADH-Ubiquinone oxidoreductase. As these complexes are proton pumping the created gradient can be used for ATP generation. *An2*) Electrons and protons are transferred simultaneously without creating a membrane potential.

Aim of this work is to present a useful analysis tool, which is able to identify beneficial production processes for microbial electrosynthesis, and at the same time enables insight into the energy conservation possibilities during anaerobic electrically enhanced fermentation. Using *in silico* approach to calculate the metabolic impact of different electron transport routes during electrically enhanced fermentation enables the evaluation of different mechanisms while current knowledge gaps remain. Pandit et al. recently presented a first computational approach that characterized the general role of bio electrosynthesis in chemical production using a genome scale metabolic model of *E. coli* [24]. Within their model it is assumed that cathodic electrons enter the metabolism and directly reduce NAD^+^ to NADH. Analogous to the theory discussed before the authors precariously assume the creation of a proton motive force that drives ATP synthesis even though the fumarate reductase of *E. coli* is, unlike the one of *A. succinogenes,* a non-proton-pumping enzyme [27]. Not surprisingly they report an increase of ATP yield caused by electron supply. We regard extracellular electron transport coupled and uncoupled to ATP synthesis and discuss the properties of both options to boost the production of various valuable products. Four different electron transport scenarios for mediated cathodic and anodic EET are described in the following paragraph and are visualised in Figure 1A and B.

### Cathode 1 (*Cat1*)

Mediator oxidation occurs on outer membrane cytochromes that transfer the electrons into the organism and finally onto NAD. Charge-imbalance creates driving force for 1 proton per electron to enter the cytosol and drive ATP-synthase (transport of 3 protons catalyses the generation of one molecule ATP).

### Cathode 2 (*Cat2*)

The mediator transfers its electrons and protons directly onto NAD without creating a driving force for ATP synthesis. This could happen by diffusion into the cytoplasm or catalyzed by enzymes such as hydrogenases.

### Anode 1 (*An1*)

Electrons from the Quinone-pool are transferred to the mediator molecule by membrane-bound cytochromes of the respiratory chain. Running the electron transport chain via Quinones creates a proton gradient that drives ATP synthesis.

### Anode 2 (*An2*)

The anode acts like an electron sink by directly accepting electrons from NADH. This could happen either catalyzed by membrane-bound enzymes such as hydrogenases or by diffusion of the mediator into the cytoplasm.

Note that apart from the mediator diffusion model all models could theoretically also happen as direct electron transfer between the electrode surface and the cellular membrane.

### Metabolic modelling by elementary mode analysis

We created core networks of metabolic carbon pathways to determine the effect of electrical enhancement through the different EET ways on production. The tool chosen for the metabolic analysis is elementary mode analysis (EMA), which determines all possible solutions of the metabolic matrix by calculating a unique set of so called *elementary flux modes* (*efm*s) [28]. Each *elementary flux mode* pictures the proposed cellular metabolism in steady-state conditions and together all *efm*s span the complete solution space for each network. Within this solution space we can determine maximum yields for certain products and reconstruct carbon fluxes within the network for example to study changes in by-product formation. The advantage of EMA over other modelling approaches is the calculation of ALL solutions rather than only one best solution (e.g. in flux balance analysis). Thereby we can not only assess theoretical maximum yields for production and biomass formation but are also able to compare all possible metabolic flux distributions, which presents a more holistic view of the impact of each EET model [29].

Elementary mode analysis is based purely on stoichiometry of the reaction equations and steady-state conditions of the organism. Therefore the solution space can be regarded as outer boundaries of the metabolic possibilities. Here we use this effectively to determine the maximum theoretical possible advantage of EET on production. Actual *in vivo* yields will lie inside the determined solutions space. However they will usually be lower than the theoretical maximum yield and will depend on many factors such as thermodynamics, enzyme kinetics, gene regulation and product toxicity, which are not taken into account here.

The presented metabolic analysis was implemented exemplarily for the central carbon metabolism of *Escherichia coli* as model organism for industrial biotechnology. Tools for its genetic modification are well established which makes it an attractive host for the production of various compounds. Even though *E. coli* does not show a comparable electrical activity to *Geobacter* or *Shewanella* species, it was shown to be able to exchange electrons with electrodes via soluble mediator molecules [30-32]. Furthermore recent studies report successful transfer of functional molecules from the electron transport chain of *Shewanella oneidensis* into *E. coli* and therefore suggest that the microbe could be modified for optimized electron exchange mechanisms [33,34].

## Results and discussion

**Figure 2:**
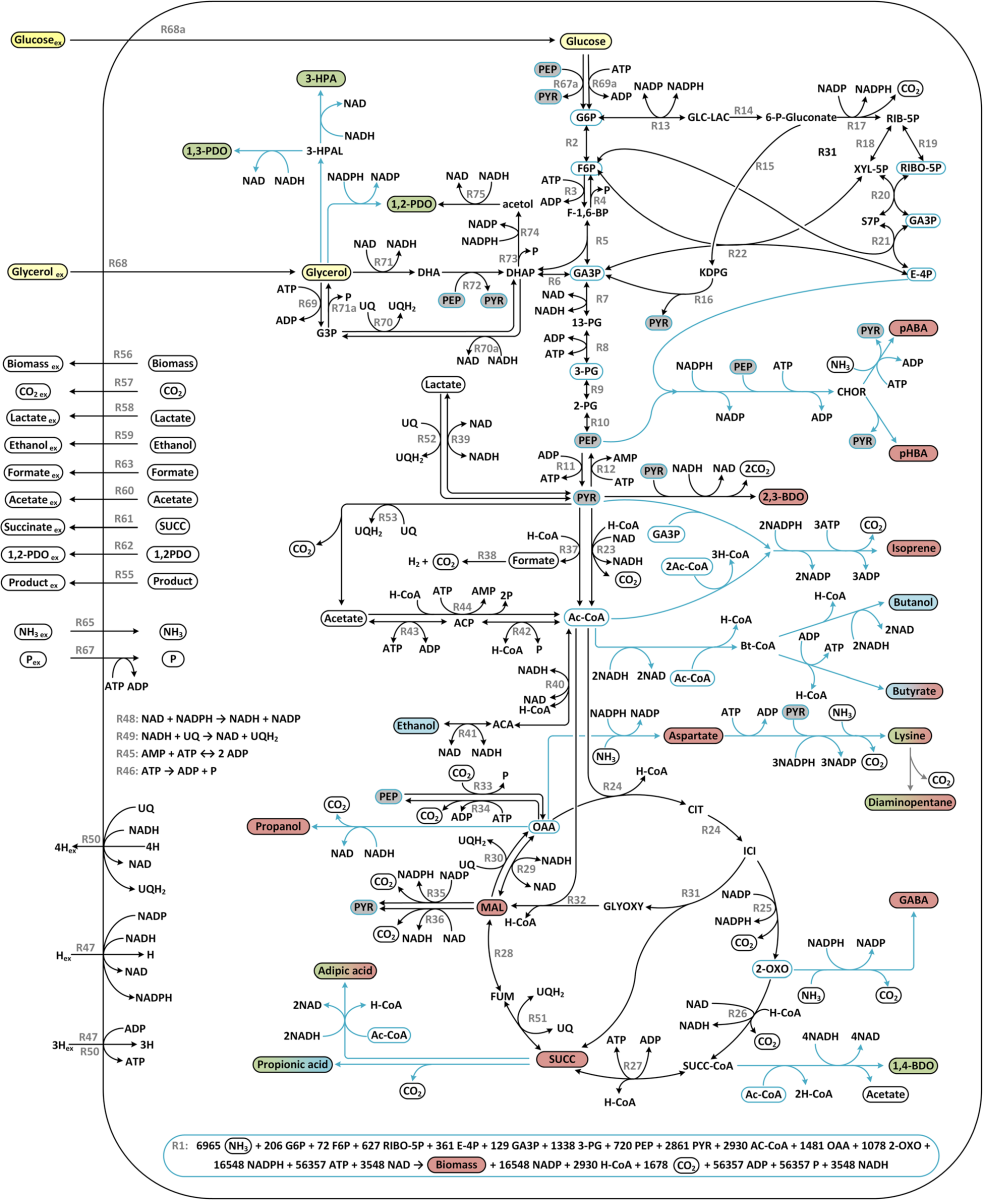
**Metabolic core network including production pathways.** Metabolic fluxmap of the *E. coli* network including exchange reactions and production pathways. All reactions of the core network are shown with their respective number, RX, as given in Additional file 1. Production pathways are condensed to single reaction steps displayed in light blue. Required precursors for biomass formation (R1) are labelled with blue borderline. The substrates are highlighted in yellow while all target products are coloured according to their most beneficial electron-exchange-option. Increased production by anodes are displayed red, increased product yields by cathodes green while no benefit from either electron transport is shown blue. If a product shows increased yields on different electrodes depending on the substrate a mixed colour pattern was chosen. Key abbreviations are given in the supplementary information.

In the following sections we present calculated carbon yields for the production of biomass and various valuable compounds including carboxylic acids, alcohols and aromatics via electrically enhanced fermentation. The initial idea of microbial electrosynthesis was to start from the fully oxidized substrate CO_2_ and provide all electrons by an electrode. But a disadvantage of using CO_2_ as sole substrate is the extremely high electron demand and energy limitation by the strictly anaerobic pathways such as the Wood Ljungdahl pathway. Hence many approaches regard the conversion of organic molecules from waste streams, such as acetate, lactate or glycerol by non-acetogenic organisms as more beneficial [35,36]. Within this work we focus on microbial electrosynthesis from substrates other than CO_2_, mainly glucose and glycerol, a process which is often referred to as “electro fermentation” [7,37]. Sugar fermentations are dominating in bio-industry and were therefore investigated to determine the potential of electrical enhancement to boost these processes [38]. As a second substrate of interest glycerol was chosen as it represents a cheap C-source often produced as a waste in biodiesel production [39]. Its more reductive state compared to glucose suggests it could result in higher yields when converted into more reduced compounds and require less additional electrons [40]. A current review by Jang et al. summarizes important C2-C6-products and their biological production [38]. We implemented all anaerobic production pathways in our metabolic network and analysed the theoretical yields of each compound under electrical enhancement. Figure 2 shows the metabolic fluxmap of the presented *E. coli* carbon network including all product pathways. A full list of all maximum product yields, with and without biomass formation as well as the number of computed *elementary flux modes* for each substrate and product combination can be found in Additional file 1.

### Impact of EET mechanism on Biomass yields

In absence of a final electron acceptor for the respiratory chain the anaerobic formation of biomass is generally limited by the availability of energy and the overproduction of reduced redox equivalents [41]. This becomes clear by studying reaction R1 in Figure 2, which shows the coupling of biomass generation to ATP consumption and NADH formation. As a result an anode as electron sink increases biomass yields, especially if the energy limitation is lifted by supplying extra ATP (*An1*). On the other hand providing even more electrons through a cathode cannot significantly increase biomass yields. In fact the addition of NADH to the anaerobic network leads to considerably less elementary flux modes as the network has fewer options to distribute carbon fluxes while retaining its redox balance (see *efm* numbers in Additional file 1).

**Figure 3:**
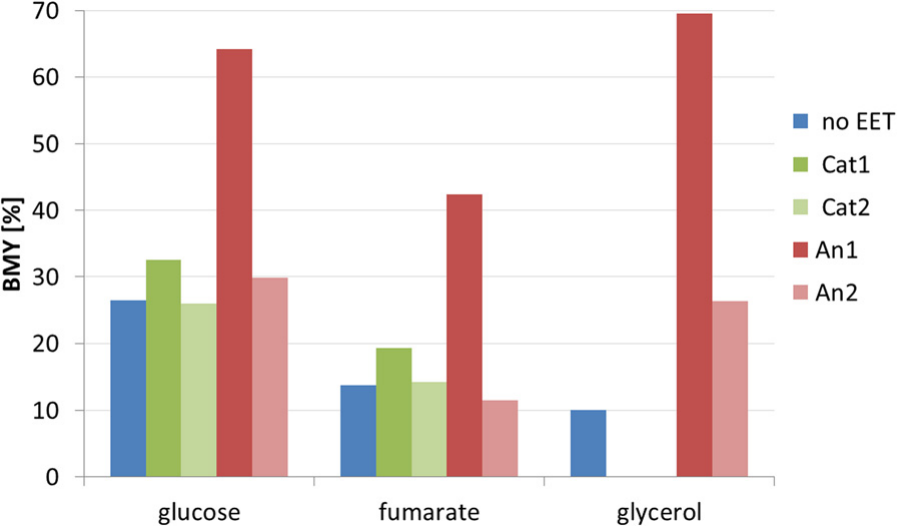
**Theoretical maximum biomass yields on different substrates with and without electrical enhancement via different electron-exchange-models.** *no EET*: no electrical enhancement, *Cat1*: cathodic electron supply coupled to energy conservation; *Cat2*: cathodic electron supply uncoupled to ATP formation, *An1*: anodic redox sink coupled to ATP generation; *An2*: anodic redox sink uncoupled to energy conservation.

The maximal carbon yields for biomass production that are achievable with the different electron transport pathways under anaerobic conditions in *E. coli* are summarized in Figure 3. For the use of glucose as substrate it can be seen that if redox power simultaneously provides additional ATP (*Cat1*) the biomass yield can be slightly increased, from maximal 26.5% to 32.5%, while the cathodic model that only supports NADH formation results in a minor yield decrease of about −0.6% (*Cat2*). The network with the anodic model *An2* acting as a pure redox sink is still ATP limited with a maximum achievable biomass yield of about 29.9%. However the anodic model that supports the creation of a proton motive force, *An1*, has the power to enhance biomass production to a maximal yield of 64.1% which equals an increase of about 37.5% and is close to the theoretical maximal biomass yield under aerobic conditions (71.5%).

The use of fumarate as substrate was investigated as it is excessively used in literature that studies enhancement of succinate production by EET [42,43]. This includes also the studies by Park and Zeikus, which report for the first time the support of growth by electron supply through a cathode [25,26]. As discussed in the introduction this might be a unique effect of the enzyme properties of *A. succinogenes*. Activity of its fumarate dehydrogenases complex creates a proton motive force and therefore ATP synthesis while this cannot be translated to other organisms such as *E. coli* [27]. The maximal biomass yield for *E. coli* grown anaerobically on fumarate was determined to be 13.6% for non-enhanced conditions while cathodic EET causes an increase to 19.3% and 14.2% for *Cat1* and *Cat2,* respectively. To create the amounts of ATP and NADPH needed for biomass formation from fumarate the metabolism is required to produce NADH. Without EET the NADH is mainly produced by the malic enzyme and pyruvate decarboxylase so that in both cases one carbon is “lost” in the form of CO_2_. NADPH is also created by a malic enzyme under CO_2_ release (Figure 2). The ATP demand is fulfilled by a combination of running the electron transport chain with NADH as electron donor and fumarate as final electron acceptor and the acetate producing acetate kinase. This leads to a maximal possible biomass yield of 13.6% with the main by-products succinate (55%), CO_2_ (19%) and acetate (13%). The assumption that cathodic EET results in an increase of available NADH (*Cat2*) reduces the by-product spectrum to carbon dioxide and succinate only. With fumarate as final electron acceptor the electrons from NADH can enter the first step of the electron transport chain and create a proton motive force, which can drive the highly efficient ATPase. NADPH is created by the membrane bound transhydrogenase driven by proton gradient. This reaction consumes 0.33 ATP equivalents per transhydrogenation (see reaction R47 in Figure 2 and Additional file 1). This results in a maximal biomass yield of 14.2% for *Cat2* with 6% CO_2_ and 80% succinate as by-products. The extra ATP available in case of *Cat1* results in a biomass yield of maximal 19.3% with 9% CO_2_ and 72% succinate. The high succinate formation in all cases points out that fumarate might be an interesting substrate to study electron transport but it is not considered a feasible feedstock for bio-processes due to availability, price and the considerable amount of succinate as a by-product that is to be expected (see above).

Biomass yields calculated for growth on a further reduced substrate such as glycerol cannot be improved by providing additional electrons or protons as the breakdown of glycerol is highly limited by the availability of an electron acceptor. Usually anaerobic growth with glycerol as sole substrate is coupled to the production of hydrogen or 1,3-propanediol as this includes pathway branches that consume NADH created during biomass formation [40,44]. If the cellular NADH level is further increased, redox balance can no longer be obtained and growth is inhibited. Still, growth on glycerol with additional electron uptake by a cathode is possible if it is coupled to a production pathway that balances NADH (e.g. propanediol and butanediol see following part of this work). *An2* increases the maximal achievable biomass yield on glycerol from 10.0% to 26.4%. Again *An1* results in a major increase of the max biomass yield up to 69.5% by providing additional ATP.

The here presented metabolic benefits of increased ATP availability and improved redox balance offered by an anode might be an explanation for the observed thick biofilms on anodic electrodes and poor growth of cathodic cultures [15,18].

### Impact of EET mechanism on production

The decision if microbial electrosynthesis will become an important technique in bio industry will strongly depend on the product yield increase that it can trigger. Therefore it is important to understand the effects of different electron transport routes and energy conservation mechanisms that might happen during electrical enhancement. The degree of reduction (DoR) of a product is often used to describe the electron demand of its production. In fact this is only useful for a direct conversion. The DoR is calculated by the formula given in Table 1 and characterizes a molecule by its oxidative or reductive state.

**Table 1:** **Formula to calculate the degree of reduction (DoR) for substrates and products (Erickson et al.** [45]**)**

**C**_**a**_ **H**_**b**_ **O**_**c**_ **N**_**d**_ **S**_**e**_ **P**_**f**_	**C**	**+4**
$DoR=\frac{4a+1b-2c-3d+6e+5f}{a}$	H	+1
	O	−2
	N	−3
	S	+6
	P	+5

**Figure 4:**
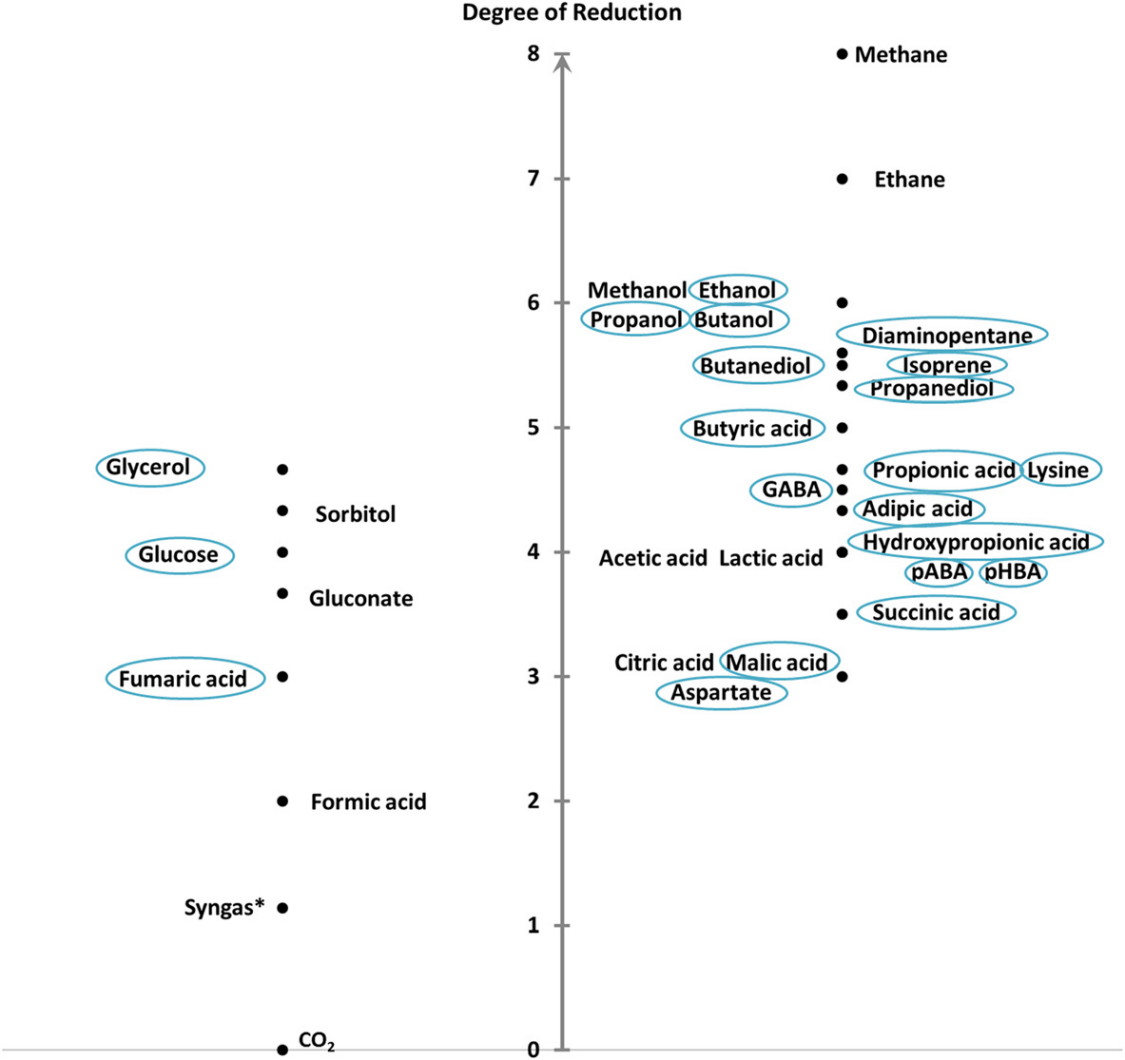
**Degree of reduction of several industrial relevant substrates (left) and products (right).** Highlighted are choices of substrates and products used in this study. *The given DoR of syngas refers to synthesis gas with an average composition of 40%CO, 30%CO_2_ and 30%H_2_.

Figure 4 shows a selection of biotechnologically important substrates and products sorted by their DoR. Starting from sugars (DoR_glucose_ = 4) one would expect a benefit from additional electron supply for the production of all compounds with a DoR higher than 4, such as primary alcohols (e.g. DoR_ethanol_ = 6) or some carboxylic acids (e.g. DoR_butyric acid_ = 5). In fact we observe an overall limited predictive power of the DoR as many products with a higher degree of reduction than the substrate show no increased yield with increasing availability of redox equivalents (e.g. ethanol). Contrary we could also find substrate-product-combinations that benefit from extracellular electron supply even though their reductive state is equal (e.g. 3-hydroxy-propionic acid from glucose). Furthermore it was observed that the production of two isomers of the same compound can benefit from opposing redox interference: While the production of 2,3-butanediol is increased in presence of an anode, 1,4-butanediol production benefits from additional electron supply by a cathode (see Additional file 1). Therefore the presented stoichiometric approach is absolutely essential to determine the actual redox balance of a microbial conversion and identify substrate-product-combinations that could benefit from EET.

Figure 2 shows several products that benefit from the presence of a cathode *and* an anode depending on the used substrate: propionic acid, butyric acid, adipic acid, lysine and diaminopentane. The different response to electrical enhancement can be explained by the use of either glucose or glycerol as carbon-source. All compounds mentioned above are derived from acetyl-CoA or intermediates of the tricarboxylic acid cycle (see Figure 2). To generate these metabolites glucose is broken down by glycolysis where glycerol only enters further downstream. At the end of glycolysis the final metabolite pyruvate is generated with equimolar amounts of NADH and ATP if derived from sugar. With glycerol as the only carbon source 2 mol NADH per mol pyruvate and ATP are created. Therefore some production pathways that re-oxidize only one NADH per pyruvate consumed benefit from an anode on glycerol while a cathode might promote production from glucose. Fully redox-balanced production pathways such as for ethanol or butanol cannot be optimized by electrical enhancement (see Figure 2).

**Figure 5:**
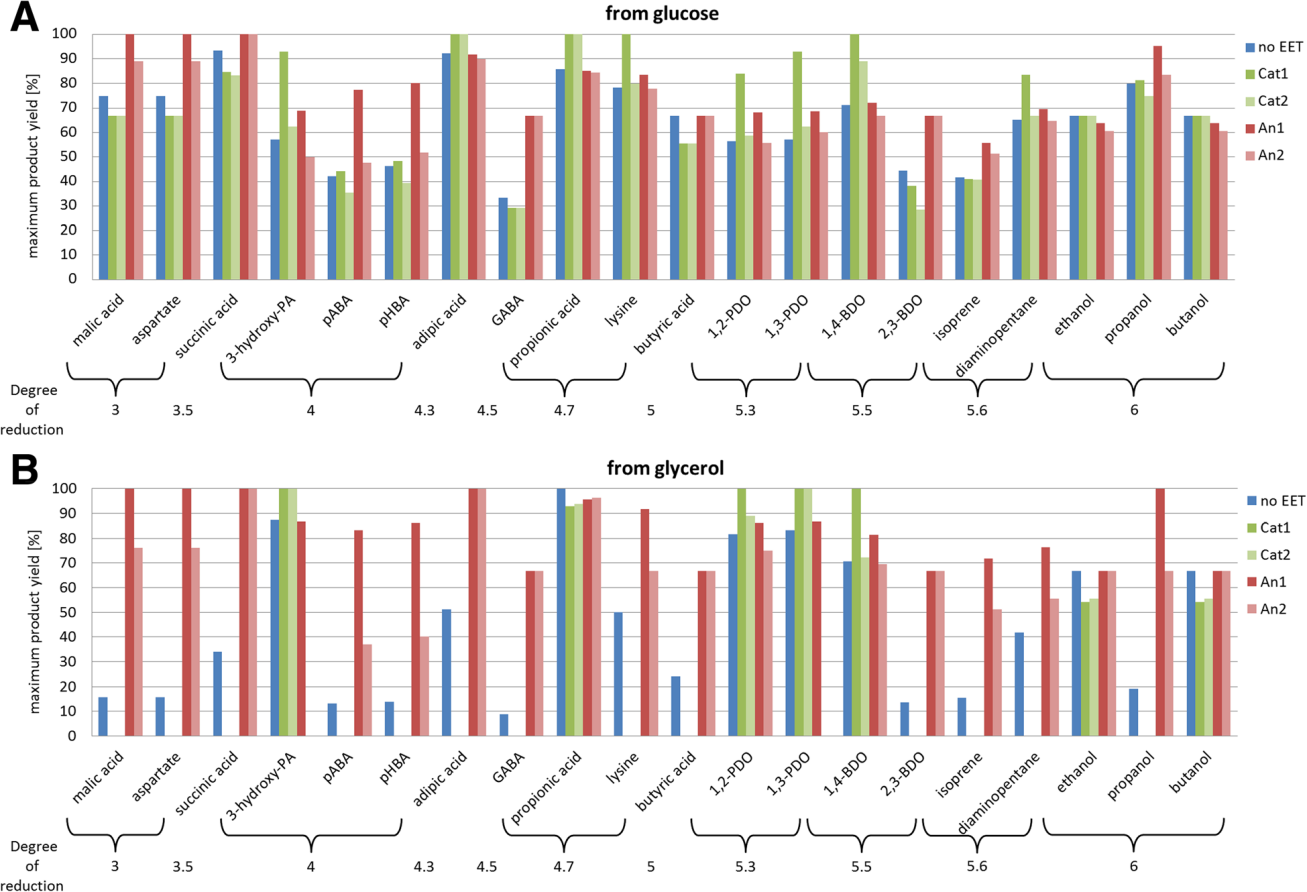
**Theoretical maximum carbon yields for different products with and without electrical enhancement. (A)** Summarizes all product yields for fermentation from glucose (degree of reduction_glucose_ = 4) while **(B)** shows the maximum yields on glycerol (degree of reduction_glycerol_ = 4.6). *no EET*: no electrical enhancement, *Cat1*: cathodic electron supply coupled to energy conservation; *Cat2*: cathodic electron supply uncoupled to ATP formation; *An1*: anodic redox sink coupled to ATP generation; *An2*: anodic redox sink uncoupled to energy conservation; BDO: butanediol, GABA: γ-aminobutyric acid, PA: propionic acid, PDO: propanediol.

The results of all calculated productions and the effects of the cathodic and anodic electron transport models are summarized in Figure 5 and Additional file 1 and are discussed in the following sections.

### Cathodic processes that promote microbial electro reduction

The elementary mode analysis could identify several carboxylic acids and alcohols that show increased production from glucose and glycerol under extracellular electron supply by a cathode. Propionic acid and adipic acid are both derived from the tricarboxylic acid cycle intermediate succinate and show maximum achievable carbon yields of 100% on a cathode if produced from glucose (see Figure 5A and Additional file 1). Propionic acid is conventionally produced from petro chemicals and has many applications of industrial scale including food additives, perfumes, and pharmaceuticals. Sustainable microbiological production is most promising in natural producers such as *Propionibacterium* that are able to ferment a variety of carbon sources [46,47]. It could be shown that the use of glycerol is advantageous as its more reduced state compared to hexoses and pentoses benefits the overall metabolic redox state resulting in higher product yields and reduced by-product formation [48]. Accordingly our metabolic analysis determined the maximum achievable yield of propionic acid from glycerol to 100% (see Additional file 1). We propose that for glucose fermentation this maximum yield can also be achieved if additional electrons are provided by an electrode. The last step of propionic acid production in *E. coli* is the decarboxylation of succinate which results in a “loss” of one carbon atom in CO_2_. Additional redox power allows the recirculation of CO_2_ by the oxaloacetate forming phosphoenolpyruvate carboxylase as the increased availability of NADH enables formation of malate and therefore succinate and PA (see Figure 2). The mechanism by which electrons are fed into the metabolism seems subordinate as for both cathodic models the max yield is 100%. However, comparison of the highest yields that enable simultaneous growth reveals a benefit from scenario *Cat1* where the additional ATP input enables a max product yield of 97.7% with a biomass yield of 2.1% and 0.2% CO_2_ as the only by-products. Electron supply by *Cat2* by contrast results in a maximal growth-coupled product yield of 89.6% which is not much higher than the non-enhanced production (85.3%). A full list of all maximum yields for growth coupled production of each substrate-product-combination is given in Additional file 1. Also the typical by-products of propionic acid fermentation succinate, CO_2_ and acetate are detected in more than 85% of the 1,965 *efms* of *Cat2*. Whereas none of the 2,840 *efms* that use *Cat1* produces acetate as the influx of electrons and protons results in sufficient ATP production. Emde and Schink reported similar results for *in vivo* fermentation of *Propionibacterium freudenreichii*, in which they observed an increased production of propionic acid in presence of reduced mediators while acetate formation was inhibited [14]. According to our calculations this would be an indication towards an EET mechanism as proposed for *Cat1* since the shift in product spectrum suggests that the cathode supports an alternative ATP source to acetate production. These results demonstrate that electrical enhancement could be a suitable technique to boost propionic acid production from glucose by reducing by-product formation. But they also highlight the importance of unveiling the actual connection between extracellular electron transport and energy metabolism for the viability of microbial electrosynthesis.

The second group of products that benefit from additional electron supply by a cathode are compounds derived directly from glycerol or the upper branch of glycolysis such as 3-hydroxypropionic acid, 1,2-propanediol and 1,3-propanediol (Figure 2). 1,2- and 1,3-propanediol (1,2-PDO; 1,3-PDO) are a building blocks for polyesters and even though mostly produced chemically from propylene oxide and propenal, respectively, there are several emerging approaches for their microbiological production. DuPont Tate and Lyle BioProducts have already commercialized several corn-sugar-based 1,3-PDOs (http://www.duponttateandlyle.com). Reported yields of these glucose based fed-batch fermentations with engineered *E. coli* are around 60% [49-51], which is close to the theoretical maximum product yield on glucose that we computed for a non-enhanced network (57.1%). Our calculations suggest the maximum yield of this process (which is already on the commercial market!) could be increased up to 92.9% by electrical enhancement (see Figure 5A and Additional file 1). But the benefit achievable with bioelectrochemical techniques for propanediol production is strongly dependent on the actual EET mechanisms. The product yield during anaerobic glucose fermentation is not only redox but also energy limited. The production of 1,3-PDO from both feedstocks can be summarized with equations 1 and 2. The usage of glucose requires twice the amount of reducing equivalents and also a high energy phosphate bond (~P_i_) such as ATP or PEP for the phosphorylation of sugar.

1. ½ glucose + ~P_i_ + 2 NADH + 2 H^+^ → 1,3-PDO + H_2_O + 2 NAD^+^ + P_i_,
2. glycerol + NADH + H^+^ → 1,3-PDO + H_2_O + NAD^+^.

**Figure 6:**
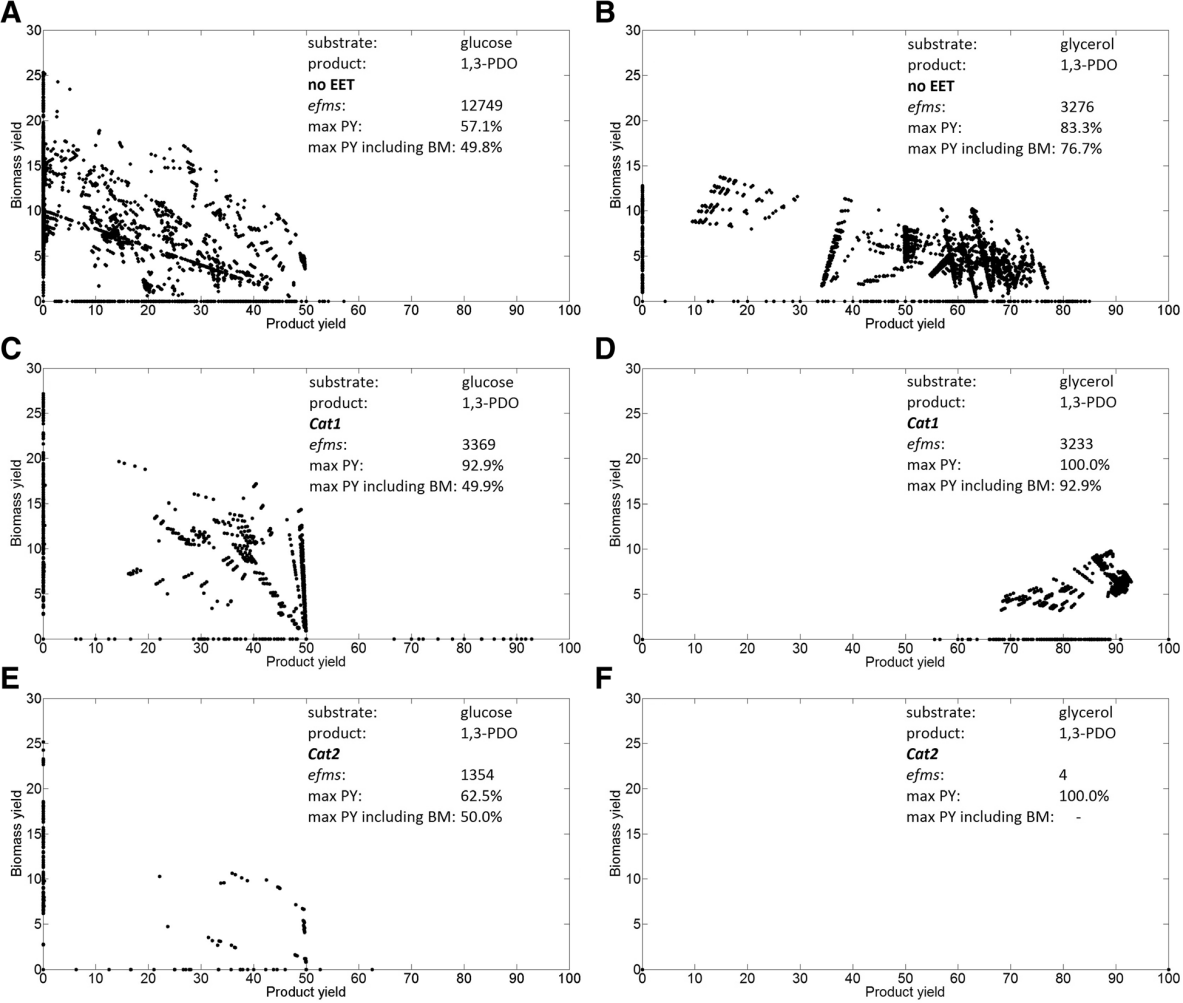
**Plots of biomass and product yields of all elementary flux modes for anaerobic 1,3-propanediol production.** Each data point in a plot represents the biomass and propanediol yield for a calculated elementary flux mode that uses the respective electron transport model. Graph **A**, **C** and **E** utilize glucose as substrate under different electrical conditions. Graph **B**, **D** and F utilize glycerol as substrate under different electrical conditions. Text inserts in each graph summarize the corresponding substrate and product, electron transport model, number of computed elementary flux modes (*efms*) and maximum theoretical product yields (PY) with and without biomass (BM) production. *no EET*: no electrical enhancement, *Cat1*: cathodic electron supply coupled to energy conservation; *Cat2*: cathodic electron supply uncoupled to ATP formation.

Therefore *Cat1* which provides simultaneously NADH and ATP causes a significant increase of the maximal 1,3-PDO yield to final 92.9% for glucose. If *Cat2* would represent the dominant mechanism the product yield could only slightly be increased to maximal 62.5% as the energy limitation would still remain. The production of 1,3-PDO from glycerol also benefits from electron supply by a cathode as seen in Figure 5B. Here both cathodic models result in a maximum yield of 100%, yet the impact on the possible operational options for the network differs significantly for the different EET scenarios. Figure 6 displays the plots of biomass against product yields for each *elementary flux mode* during production of 1,3-PDO. The transfer of electrons into the cellular metabolism via cytochromes (*Cat1*) displays for both substrates the most beneficial option (Figure 6C and D). For glucose the product yield is increased significantly and for glycerol not only the maximum achievable yield is improved but also the majority of the cathodic modes features high product and low biomass yields. The production pathway is the most efficient option for the network to maintain cellular redox balance as the internal NADH level is increased. This way cathodic electron supply is automatically coupled to product formation. For the production of PDO from glycerol all modes that take up electrons via *Cat1* have product yields above 55%. Moreover 91% of the total 3,233 modes couple production to biomass formation, which would enable production during the electrochemically enhanced fermentation (Figure 6D). For substrate-product combinations that show this behaviour, electrochemical techniques could offer the possibility to force the metabolism to operate in a desired mode(s) comparable to genetic engineering approaches. But also operational modes that show increased product yields without growth, offer an interesting perspective for microbial electrosynthesis. For the fermentation from glucose it can be seen that all modes above 50% PDO-yield do not produce any biomass (*Cat1*) (Figure 6C). This is also the case for both top modes (from glucose and from glycerol) with 100% product yield. The flux distributions of these particular networks show a carbon flux that could be titled as “true catalysis”. The substrate is converted directly into the product by the addition of redox power from the electrode while no by-products are created. To realise “true catalysis” a two-part fermentation strategy could enable successful production by coupling a non-enhanced growth phase to a later electrically supported production phase.

### Anodic processes promote microbial electro oxidation

The use of an anode in bio-electrochemical systems was widely studied in the research of microbial fuel cells, which create electricity as their main product. Substrates are usually mixed waste streams but also glucose and glycerol containing media are used [52-54]. Since the power output of these systems is too small to become relevant on industrial scale any time soon and because of the inherently low value of electricity, the focus shifts towards higher value products [8,55]. We identified several valuable compounds that show increased production in presence of an anode (Figure 5 and Additional file 1). It can be seen that without electrical enhancement yields on glycerol are generally a lot lower than on glucose. This is due to the surplus of NADH created during glycerol degradation, which anaerobically becomes limiting if there are no NADH consuming pathway branches such as PDO production [39]. In presence of an anode as electron sink the cellular redox state is optimized and product yields on glycerol can be increased to similar levels as on glucose. Products that benefit from anodic electron transport include glycolysis derived products (isoprene, 2,3-butanediol), products from the tricarboxylic acid cycle (malate, succinate) and its derivatives (propanol, aspartate, γ-aminobutyric acid) and aromatics from shikimate pathway (*para*-aminobenzoic acid, *para*-hydroxybenzoic acid) (see Figure 2). Again the actual EET mechanism is of great importance as the anode that promotes ATP synthesis (*An1*) triggers significant higher yield increases in many cases (see Figure 5).

2,3-butanediol (2,3-BDO) is an interesting example to study as the production is increased by an anode even though the substrates glucose and glycerol are further oxidized (DoR_2,3-BDO_ = 5.5, DoR_glucose_ = 4; DoR_glycerol_ = 4.7). It also shows reverse behaviour to its isomer 1,4-butanediol that benefits from a cathode, which is due to the different production pathways (see Figure 2 and Additional file 1). 2,3-BDO has applications in the food, pharmaceuticals as well as agrochemical markets and is still produced from fossil fuel feedstocks [38]. But there are several promising approaches for its microbiological production from sugars or glycerol [56]. It is metabolically derived from pyruvate via acetolactate and the recent progress in process optimization suggests bio-2,3-BDO will hit the industrial market soon [56,57]. The degradation of glycerol and glucose creates in both cases equimolar ratios of reducing equivalents and pyruvate. Because the production pathway of 2,3-BDO requires only one NADH per two molecules pyruvate an overall surplus of NADH is accumulated. An anode as electron sink can help to optimize NADH/NAD ratios and reduces the formation of by-products such as lactate or ethanol which are otherwise used as electron sink. In this case the transport mechanism of electrons is subordinate as the limitation is purely stoichiometric. Increasing ATP levels cannot improve the maximum yield which is constrained by CO_2_ formation due to decarboxylation steps during production. Therefore the maximum theoretical yield for 2,3-BDO production on an anode (*An1* and *An2*) is 66.7% from glucose and glycerol, respectively (Additional file 1).

However, many compounds are not solely redox limited in their production pathways and therefore the coupling of electron transport to energy conservation is of major importance as was shown before for 1,3-PDO production on a cathode. The here studied production of malic acid, propanol, isoprene, aspartate, *para*-aminobenzoic acid (pABA) and *para*-hydroxybenzoic acid (pHBA) on an anode benefits strongly from the proposed mechanism *An1* whereas a pure NADH-redox-sink (*An2*) results in a significantly smaller yield increase (full table of theoretical yields for all products and EET models is given in Additional file 1). The biggest difference of the two electron transport models is seen for production of the aromatics pHBA and pABA. These are used in sunscreens, dyes, liquid crystal polymers, polyurethanes and food additives and have also the potential to act as building blocks for aromatic polymers [58]. Even though purely synthesised from petro chemicals to date, there is potential for the bio-production of pHBA and pABA as microbes such as *E. coli* produce the aromatics via the shikimate pathway. This pathway requires phosphorylated Co-factors such as NADPH, ATP and PEP and therefore shows major possible flux increases by *An1*. pABA yields could theoretically be increased from 42.1% and 13.1% to 77.4% and 83.3% on glucose and glycerol, respectively. Solely the presence of an anodic electron sink by *An1* causes a shift of the theoretical maximum yield for pHBA production from 46.3% to 80.2% on glucose and 13.9% to 86.3% on glycerol (Figure 5). These promising results of redox optimisation by electrical interference could offer a new basis for metabolic engineering towards these new products.

## Conclusions

To understand benefits and limitations of microbial electrosynthesis a detailed understanding and analysis of the involved metabolic processes is needed. The presented analysis is the first published approach to methodically screen bio-production processes for their potential benefit from electrical enhancement and could successfully identify 18 target products with possible product yield increases between 7% and 84%. Even though *in vivo* yields will usually be lower than the here presented theoretical maximum yields these examples show a great potential of microbial electrochemical techniques to boost anaerobic glycerol and sugar fermentation. Contrary to the assumption reduced compounds such as bio-fuels and alcohols would always require electron input we revealed 12 production processes that show increased product yields on an anode while only 6 of the 20 studied products benefit from additional electron supply. Due to the anoxic fermentation conditions required for an electrically enhanced process intracellular NADH levels are increased, so that in most cases an overall surplus of redox equivalents is accumulated. A benefit from cathodic EET is only seen for production pathways that purely rely on NADH input (e.g. 1,2-PDO, 1,3-PDO, 3-hydroxypropionic acid) while other products even though further reduced than the substrate are limited by redox surplus and/or energy requirements (e.g. propanol, 2,3-butanediol).

The accumulation of NADH during anaerobic growth could also explain the poor growth performance observed for cathodic cultures. Our analysis shows that the presence of an anode promotes biomass formation while electron supply by a cathode limits the metabolic options of the organism during growth. This limitation could possibly be turned into a benefit by coupling growth to production as explained for the example of the anaerobic conversion of glycerol to 1,3-PDO on a cathode.

The elementary mode analysis does not only identify target processes but also highlights the major importance of electron transport mechanism and its coupling to energy conservation. For the majority of products a crucial dependence of maximum achievable product yield and ATP availability was detected. Therefore it is important to direct the focus of current research in the microbial electrosynthesis community towards fundamentals of electron transport as these are needed to be understood to design processes that approach the full potential of microbial electrochemical techniques.

## Methods

The basic *E. coli* core model used for the *in silico* analysis includes: Embden–Meyerhof–Parnas pathway/glycolysis, glycerol degradation, Entner–Doudoroff pathway, pentose phosphate way, tricarboxylic acid cycle, glyoxylic shunt, anaplerotic reactions, anaerobic fermentation, electron transport chain, import and export reactions and interaction with a soluble electron carrier (see Figures 1 and 2). For the production of industrial relevant products that are not metabolites of the main network, engineered pathway branches for production were implemented. Because oxygen will lead to abiotic current production in most cathodes, we assumed anaerobic conditions as a technical requirement and performed all calculations under anaerobic conditions.

The main network includes 57 metabolites, 75 Reactions (24 reversible) and we calculated up to 215,000 *efms* per scenario. The full networks can be found in the supplementary information (Additional file 1).

The stoichiometric analysis of metabolic networks was performed based on the elementary mode analysis framework introduced by Schuster et al. [59] The java implemented free software *efmtool* (version 4.7.1) [60] was used within MATLAB, MathWorks (version R2012a), to compute for each network the elementary flux modes which represent all possible and unique steady-state flux distributions the network could have based on reaction stoichiometry. It does not take regulatory or thermodynamic constraints into account, which means that the theoretical maximum yields are the absolute maximum possible and that *in vivo* yields will very likely be lower. Nevertheless, it represents the most reliable estimate of the capacity of a network. The operational mode that shows maximum possible carbon flux from substrate into product is referred to as *top mode*. Maximal theoretical yields for biomass or a certain product are obtained by calculating the carbon balance of all carbon containing substrates entering the network and the carbon containing products leaving the network:

$$ Yiel{d}_{product}\ \left[\%\right]=\frac{flu{x}_{product}\  carbo{n}_{product}}{flu{x}_{substrate}\  carbo{n}_{substrate}}100\%. $$

Where *flux*_*product*_ is the reaction rate for products leaving; *flux*_*substrate*_ the reaction rate for substrate uptake and *carbon*_*product*_ and *carbon*_*substrate*_ the number of carbon atoms in the product and substrate molecules, respectively.

## Additional file

Additional file 1:
**Metabolic networks and theoretical yields.** This file provides detailed information about the studied *E. coli* carbon networks. The used stoichiometric reactions are given for the core network as well as for all production and electron transport pathways. Furthermore all computed theoretical maximum yields for each substrate product combination and EET scenario are listed including maximum growth-coupled product yields and number of *efms*.

## Abbreviations

2,3-BDO: 2,3-Butanediol
DoR: Degree of reduction
EET: Extracellular electron transfer
*efm(s)*: Elementary flux mode(s)
pABA: *para*-Aminobenzoic acid
1,2-PDO: 1,2-propanediol
1,3-PDO: 1,3-propanediol
pHBA: *Para*-Hydroxybenzoic acid
